# An Aqueous Ca‐Ion Battery

**DOI:** 10.1002/advs.201700465

**Published:** 2017-10-26

**Authors:** Saman Gheytani, Yanliang Liang, Feilong Wu, Yan Jing, Hui Dong, Karun K. Rao, Xiaowei Chi, Fang Fang, Yan Yao

**Affiliations:** ^1^ Department of Electrical and Computer Engineering and Materials Science and Engineering Program University of Houston Houston TX 77204 USA; ^2^ Department of Materials Science Fudan University Shanghai 200433 China; ^3^ Department of Chemical and Biomolecular Engineering University of Houston Houston TX 77204 USA; ^4^ Texas Center for Superconductivity at the University of Houston Houston TX 77204 USA

**Keywords:** aqueous‐based batteries, Ca‐ion batteries, copper hexacyanoferrate, polyimide

## Abstract

Multivalent‐ion batteries are emerging as low‐cost, high energy density, and safe alternatives to Li‐ion batteries but are challenged by slow cation diffusion in electrode materials due to the high polarization strength of Mg‐ and Al‐ions. In contrast, Ca‐ion has a low polarization strength similar to that of Li‐ion, therefore a Ca‐ion battery will share the advantages while avoiding the kinetics issues related to multivalent batteries. However, there is no battery known that utilizes the Ca‐ion chemistry due to the limited success in Ca‐ion storage materials. Here, a safe and low‐cost aqueous Ca‐ion battery based on a highly reversible polyimide anode and a high‐potential open framework copper hexacyanoferrate cathode is demonstrated. The prototype cell shows a stable capacity and high efficiency at both high and low current rates, with an 88% capacity retention and an average 99% coloumbic efficiency after cycling at 10C for 1000 cycles. The Ca‐ion storage mechanism for both electrodes as well as the origin of the fast kinetics have been investigated. Additional comparison with a Mg‐ion cell with identical electrodes reveals clear kinetics advantages for the Ca‐ion system, which is explained by the smaller ionic radii and more facile desolvation of hydrated Ca‐ions.

The rapid growth of the integration of renewable energy sources to the electricity grid to improve its cleanness and efficiency has urged extensive research in electrochemical energy storage technologies.^1^ In large‐scale grid storage, cost, high‐rate performance, and safety are relatively more important criteria compared to the energy density which is the key factor in portable electronic devices.^2^ In the path toward exploring a reliable energy storage system for large‐scale applications, multivalent‐ion batteries particularly Mg‐ and Al‐ion batteries have attracted a lot of interest.^3^ Multivalent ions can transport more electrons per ion giving a similar volumetric energy density to monovalent ions by less ion storage per formula. In addition, these elements are earth abundant and are promising for developing cost‐effective storage systems.^4^ However, multivalent ions typically have higher charge densities than those of monovalent ions (Table S1, Supporting Information), and the resulting high polarization strength leads to strong binding between the ions and the negatively charged host lattice and sluggish solid‐state diffusion.^5^ In contrast to Mg‐ and Al‐ion, Ca‐ion has charge density and polarization strength similar to those of Li‐ion thanks to its relatively large ionic radius.^6^ A Ca‐ion battery may, therefore, avoid the kinetic problems commonly related to multivalent chemistries and make a promising energy storage technology.

Most previous studies on Ca‐ion batteries have been performed in nonaqueous electrolytes. The compatibility between calcium metal and the electrolyte solution is problematic.^7^ Reversible calcium deposition/stripping is only possible at a high temperature of 100 °C, and the coulombic efficiency of up to ≈16% has room for improvement.^8^ There is no other viable anode material known for Ca‐ion storage.^9^ There is more success for cathode development, with several layered oxides and Prussian blue analogues showing reversible storage.^10^ However, reasonably stable cycling performance is possible only when the host structures or electrolytes contain a certain amount of water, which is not compatible with calcium metal.^11^ The shielding of Ca‐ion by the polar water molecules is so critical that the only long‐term stable Ca‐ion storage is demonstrated in an aqueous electrolyte.^12^ These results appear to us that an aqueous battery system would be a convenient way to take advantage of the Ca‐ion chemistry.

Here, we report a fast and stable Ca‐ion battery by combining an aqueous electrolyte, a fast and highly reversible organic polyimide anode, and a high‐potential open‐framework cathode (**Figure** **1**). The aqueous electrolyte properly shields Ca‐ions and renders them less polarizing in addition to making the battery safe.^13^ The use of a polyimide anode is inspired by our recent finding of cation‐independent storage in organic carbonyl compounds in neutral aqueous electrolytes.^14^ The polyimide poly[*N*,*N*′‐(ethane‐1,2‐diyl)‐1,4,5,8‐naphthalenetetracarboxiimide] (PNDIE) stores Ca‐ion at −0.45 V versus Ag/AgCl with a specific capacity of ≈160 mAh g^−1^. We chose copper hexacyanoferrate (CuHCF) as a cathode that has a higher potential (0.7–0.8 V vs Ag/AgCl) than those of previously reported Ca cathodes (Figure S1, Supporting Information).^15^ The combination of the three gives a rocking‐chair‐type aqueous Ca‐ion battery operating at 1.2 V with excellent stability. We have investigated the storage mechanism and electrode kinetics of both the PNDIE and CuHCF electrodes with electroanalytical and structural characterization techniques. We have further compared the kinetics of Ca‐ and Mg‐ion in both the electrolyte and electrodes and justified the superiority of the Ca chemistry.

**Figure 1:**
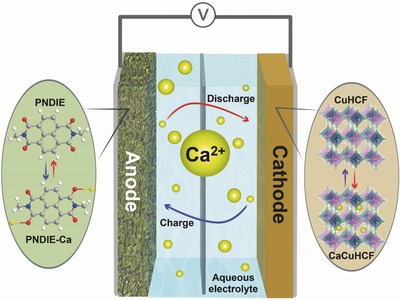
Schematic illustration of the polyimide–Ca*_x_*CuHCF aqueous rechargeable Ca‐ion battery.

PNDIE was prepared by a dehydration condensation reaction according to the method previously reported.^16^ Figure S2 (Supporting Information) shows the synthesized PNDIE powder as aggregated particles with an average size of less than 1 µm. CuHCF nanoparticles were synthesized by a simple room‐temperature coprecipitation method of mixing two aqueous transition‐metal solutions.^17^ All experiments, characterization, and electrochemical analysis are explained in detail in the Supporting Information.

**Figure** **2**a shows the charge–discharge profile for PNDIE electrode in a 2.5 m Ca(NO_3_)_2_ aqueous solution at a current rate of 183 mA g^−1^ (1C). It shows that PNDIE electrode undergoes a reversible discharge and charge process in this electrolyte delivering ≈148 mAh g^−1^ capacity between −0.9 and 0 V versus Ag/AgCl. The reduction and oxidation processes of the conjugated carbonyl groups processes are accompanied by the coordination of cations to the carbonyl groups.^18^ To have a better understanding of the redox mechanism, the changes in bonding in the reduced and oxidized states were characterized by Fourier transform infrared (FTIR) analysis. As shown in Figure 2b, the pristine PNDIE electrode shows the characteristic absorption bands at 1697 and 1660 cm^−1^ assigned to asymmetric and symmetric stretching vibrations of imide C=O bonds (ν_C=O, as_ and ν_C=O, s_), respectively.^19^ The absorption bands at 1385 and 1350 cm^−1^ are attributed to the stretching vibration of imide C—N groups (ν_C—N_). The bands located at 763 and 711 cm^−1^ are ascribed to the deformation vibration of imide C=O bonds (δ_C=O_). The peak intensity of the C=O bonds obviously decreased when PNDIE was reduced to −0.9 V versus Ag/AgCl, indicating the reduction of the double bond. The peaks do not completely disappear, however, as only two C=O are reduced at the fully reduced state.^20^ These peaks also show a slight shift to higher wavenumbers, which is the result of electron injection after the reduction reaction of C=O groups. During this reduction reaction, C—N bonds become weaker as characterized by the slight shift of the absorptions of the C—N bonds at 1350 and 1384 cm^−1^ to lower wavenumbers. In the reversed oxidation process, the absorption bands of the C=O and C—N groups fully recovered their original positions and intensities, implying a reversible rearrangement of the chemical bonds. This two‐step redox reaction exhibits two sloped plateaus within the voltage window corresponding to the utilization of two of the carbonyl groups as active redox sites. As is depicted in Figure 2c, each reduction step to form radical anion and dianion is accompanied by Ca‐ion coordination to the enolate groups with a minimum change and damage to the framework of the molecules due to the charge re‐distribution within the conjugated aromatic molecules.^19^

**Figure 2:**
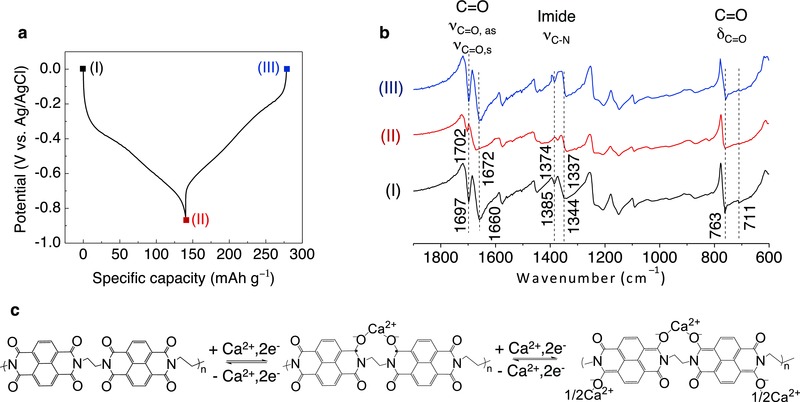
The mechanism study of PNDIE in an aqueous Ca‐ion electrolyte 2.5 m Ca(NO_3_)_2_. a) Voltage profile of PNDIE at 1C current rate. b) FTIR spectra of PNDIE electrodes recorded at different potentials as indicated in the charge/discharge profile shown in (a). c) Possible reversible electrochemical redox mechanism of PNDIE electrode.

It is necessary to point out that due to the coexistence of proton and Ca‐ion in the electrolyte, there is a possible competition between the two cations in coordination to the enolate groups during carbonyl reduction. To clarify the identity of the stored cation species, we measured the charge–discharge voltage profiles of PNDIE in aqueous electrolytes with pH equals to 3, 5, 7, and 9 (**Figure** **3**a). Except for pH = 3 where the ending of the reduction profile is not as sharp due to the higher potential for hydrogen evolution reaction, all voltage profiles overlap. These results indicate that the reduction potential of PNDIE electrode does not depend on the pH of the electrolyte, hence Ca‐ion instead of proton is the cation being stored.^14^ It is also worth noting that PNDIE provides a large overpotential for hydrogen evolution reaction (HER).^21^ This effect leads to the high average coulombic efficiency of ≈99% even at a low current rate of C/2 (Figure S3, Supporting Information). As displayed in Figure 3b, PNDIE electrode shows an extraordinary cyclic stability and ≈80% capacity retention (from 130 to 105 mAh g^−1^) and >99% coulombic efficiency after 4000 cycles at 5C current rate.

**Figure 3:**
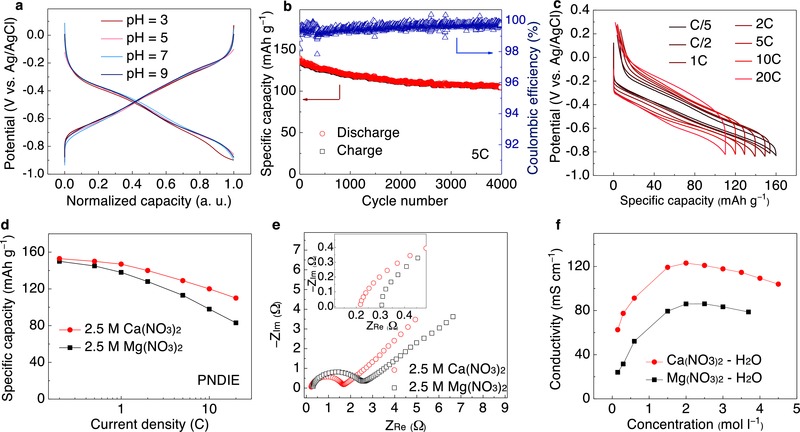
The electrochemical analysis of PNDIE in 2.5 m Ca(NO_3_)_2_ aqueous electrolyte. a) Charge–discharge profile of PNDIE electrodes in electrolytes with different pHs. b) Capacity stability and coulombic efficiency of PNDIE at 5C current rate (925 mA g^−1^). c) Galvanostatic charge–discharge profiles for PNDIE at varying current rates from C/5 (37 mA g^−1^) to 20C (3700 mA g^−1^). d) Discharge specific capacity versus C‐rate (C/5–20C) for PNDIE electrode in both 2.5 m Ca(NO_3_)_2_ and 2.5 m Mg(NO_3_)_2_ electrolytes. e) EIS spectra of the PNDIE electrode measured at 50% charge for each of the electrolytes. Inset: enlarged EIS spectra at the high‐frequency region. f) Plots of conductivity (*k*) versus salt concentration for Ca(NO_3_)_2_ and Mg(NO_3_)_2_ aqueous solutions.

In order to further investigate the kinetics of PNDIE electrode in Ca electrolyte, CV measurements at different scanning rates were measured (Figure S4, Supporting Information). The relationship between the current density and scanning rate can be expressed as *i*_p_ = *aν^b^*, where *i*_p_ is the peak current (A), ν is the sweep rate (mV s^−1^), and *a* and *b* are constants. The *b* value of 0.5 generally indicates a diffusion controlled process, while a value of 1.0 suggests that the reaction is a surface charge‐transfer process. This *b* value can be calculated from the slope of the log*i*_p_ versus logν plots. For sweep rates ranging from 0.5 to 40 mV s^−1^, the *b* values for cathodic and anodic peaks are fitted as 0.85 and 0.80, respectively, indicating an interplay between surface‐ and diffusion‐controlled reactions but predominantly a surface one.^22^ This pseudocapacitive behavior of PNDIE electrode leads to a high rate capability (Figure 3c). As the current rate increases from C/5 to 20C, the typical PNDIE plateau is maintained with the average discharge potential being −0.4 V versus Ag/AgCl.

We have also compared the rate capability of PNDIE electrode in aqueous Ca and Mg electrolytes and the results are displayed in Figure 3d. In Ca electrolyte, ≈72% of charge capacity was retained when increasing the rate from C/5 to 20C, while that in Mg electrolyte is ≈55% with a larger polarization in voltage profile (Figure S5, Supporting Information). In order to investigate the superior kinetics of PNDIE electrode in Ca electrolyte, the electrochemical impedance spectroscopy (EIS) was carried out after the cell was held at the 50% reduced state for 1 h and the Nyquist plots for PNDIE electrode in both electrolytes are shown in Figure 3e. In Ca electrolyte, the semicircle in the high‐to‐medium frequency region is smaller than that observed in Mg electrolyte, indicating faster charge‐transfer kinetics in the Ca case. The interception at the real axis at high‐frequency region arising from the ohmic resistance of the cell is also lower for the Ca cell.^23^ Both differences may be attributed to the faster diffusion of solvated Ca‐ion than Mg‐ion. The electrolytic conductivity for both Ca(NO_3_)_2_ and Mg(NO_3_)_2_ solutions at room temperature (25 °C) is shown in Figure 3e. Over the whole range of concentration tested, the conductivity of Ca(NO_3_)_2_ is consistently higher than that of Mg(NO_3_)_2_. The smaller ionic radius of hydrated Ca‐ion is responsible for the higher conductivity, which is, in turn, the result of the lower charge density Ca‐ion.^24^ The more mobile Ca‐ion in the solution leads to a lower ohmic resistance in the electrolyte and more efficient charge transfer in the PNDIE electrode where a surface reaction is dominated.

For a Ca‐storage cathode, we have synthesized CuHCF nanoparticles by a facile and easily scalable coprecipitation method described previously.^17^ This procedure creates aggregated polydispersed nanoparticles of crystalline CuHCF ranging from 30 to 100 nm in diameter (Figure S6, Supporting Information). The chemical composition of the as‐synthesized CuHCF is determined to be K_0.02_Cu[Fe(CN)_6_]_0.66_⋅3.7H_2_O by combining energy‐dispersive X‐ray spectroscopy (EDX) and thermogravimetric analysis (TGA) techniques (Figure S7 and Table S2, Supporting Information). The deviation from the nominal formula (KCuFe(CN)_6_) is attributed to the formation of a high number of disordered ferricyanide vacancies due to the fast precipitation of Prussian blue compounds in water.^17^ The obtained formula also suggests that the CuHCF is synthesized in the nearly fully oxidized state.

The CuHCF electrodes were calciated by means of a cathodic current in a 2.5 m Ca(NO_3_)_2_ aqueous electrolyte. The insertion of Ca‐ions instead of protons in CuHCF is confirmed by EDX for Ca*_x_*CuHCF samples prepared at different states of charge (SOC) and depth of discharge (DOD) (labeled A through F as shown in **Figure** **4**a). The Fe/Cu ratios for states A–F are all around 0.66, while the Ca/Cu ratio decreases linearly with the increase of SOC and then increases with DOD. The maximum Ca/Cu ratio is 0.29 corresponding to ≈0.6e^−^ transfer or ≈0.3Ca^2+^ insertion/extraction per formula of K_0.02_Cu[Fe(CN)_6_]_0.66_⋅3.7 H_2_O. This value is close to the 0.3 calculated based on the capacity of 58 mAh g^−1^ at a C/5 rate (Figure 4a). These observations confirm the insertion and extraction of Ca‐ion into and from the Ca*_x_*CuHCF structure during discharge and charge processes, respectively.

**Figure 4:**
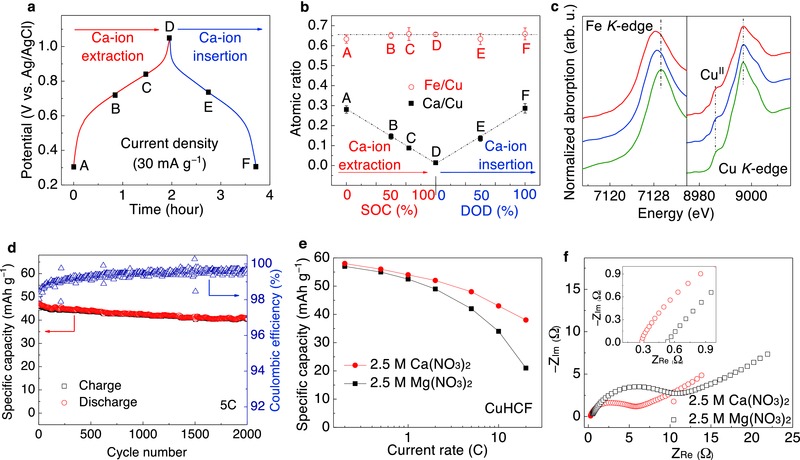
Structural and electrochemical characterization of CuHCF cathode material. a) Charge–discharge voltage profiles (vs Ag/AgCl) of the Ca*_x_*CuHCF with respect to time. Samples A–F with various Ca content at different SOCs and DODs were characterized. b) The Ca/Cu and Fe/Cu atomic ratios of samples A‐F obtained from SEM EDX elemental analyses of three different points in each sample. c) XANES spectra around Fe K‐edge and Cu K‐edge for Ca*_x_*CuHCF at different states (Bottom: fully oxidized, middle: half reduced, top: fully reduced). d) Cycling performance of Ca*_x_*CuHCF electrode at a 5C rate. e) Discharge specific capacity versus C‐rate (C/5–20C) of CuHCF electrode in both 2.5 m Ca(NO_3_)_2_ and 2.5 m Mg(NO_3_)_2_ electrolytes. f) EIS spectra of CuHCF electrode measured at 50% charge for each of the electrolytes. Inset: enlarged EIS spectra at the high‐frequency region.

Since the Ca‐ion insertion/extraction accompanies two‐electron transfer, we are interested in how the local structure changes surrounding each transition metal atoms and how each redox couple contributes to the specific capacity during Ca‐ion insertion. We carried out ex situ synchrotron radiation based X‐ray absorption near edge structure (XANES) to evaluate the valence states of Fe and Cu during Ca‐ion insertion. Figure 4b shows the XANES spectra around Fe K‐edge and Cu K‐edge for the oxidized, half‐reduced, and fully reduced samples (labeled as D, E, and F in Figure 4a). The strong main absorption peaks at around 7129 eV for oxidized sample and around 7128 eV for Ca‐inserted (i.e., reduced) samples are ascribed to the dipole‐allowed 1s to 4p transition of Fe atom. The shift of the peak from 7129.32 to 7128.27 eV during Ca‐ion insertion indicates the reduction of Fe^3+^ to Fe^2+^. This agrees with the reduction of Fe^3+^ observed for CuHCF and NiHCF during Mg insertion.^25^ In contrast, X‐ray absorption spectra around the Cu K‐edge is mostly unchanged during Ca‐ion insertion, and a pre‐edge peak at about 8986 eV corresponding to Cu^2+^ remains for all the samples.[[qv: 25a,26]] The small pre‐edge at 8982 eV for samples E and F could be explained by the ligand to metal charge transfer which suggests the strong association of inserted Ca‐ion to the strongly bonded Cu and Fe atoms by C≡N bonds.^27^ Thus, these data revealed that Fe^3+^/Fe^2+^ is the only electrochemically active redox couple during Ca‐ion insertion/extraction.

To examine the effect of calciation on CuHCF crystal structure, both CuHCF and Ca*_x_*CuHCF were investigated by high‐energy X‐ray diffraction (XRD). The diffraction peaks can be indexed to a face‐centered cubic lattice (FCC, space group Fm‐3m) (Figure S8, Supporting Information).^28^ No new peaks appeared after the calciation process, indicating an insertion mechanism through a single‐phase solid solution reaction in which the framework of CuHCF preserved after Ca‐ion insertion. However, the lattice parameter was found to decrease, as illustrated by the diffraction peaks shifting to larger diffraction angles in the fully reduced sample (Ca_0.3_CuHCF) in Figure S8 (Supporting Information). From the fully oxidized to the fully reduced state, the lattice parameter decreases from 10.27 to 10.16 Å which correlates to 1.1% strain in the crystal structure during full Ca^2+^ insertion (Table S3, Supporting Information). Similar isotropic lattice changes with charge state were previously reported for Prussian blue analogues during different cation insertions and is primarily attributed to the smaller size of [Fe(CN_6_)]^4−^ compared to [Fe(CN_6_)]^3−^ and thus the shorter length of Fe^II^—C bond compared to Fe^III^—C.^29^ This small lattice change indicates that the insertion of Ca‐ions into the CuHCF structure induces little structural distortion which results in the mechanical stability of the structure during cycling and long cycle‐life. As it is shown in Figure 4e, this electrode was cycled between 0.3 and 1.05 V versus Ag/AgCl at 5C and 88% of initial capacity retained after 2000 cycles (Figure 4d). The small decay is known to be due to the partial dissolution of the transition metals in the electrolyte which can be prevented by surface coating and adding co‐solvents into the electrolyte.^12^, ^30^

CV measurement at different sweep rates has been used to study the kinetics of CuHCF electrodes and the *b* values for both cathodic and anodic peaks were calculated as 0.74 and 0.69, respectively, indicating that the electrochemical behavior of CuHCF is a predominantly diffusion‐controlled process (Figure S9, Supporting Information). However, the open framework structure and nanoparticulate morphology of CuHCF (see Figure S6, Supporting Information) provide high ionic conductivity and short diffusion pathways within nanoparticles, allowing for rapid kinetics. When the current increases by 100 times from C/5 to 20C, 65% of specific capacity retained (Figure 4f and Figure S10, Supporting Information). This capacity retention is higher than that observed in a Mg electrolyte where a 33% of the capacity retained from C/5 to 20C. The data on impedance measurement for CuHCF in these two electrolytes can be found in Figure 4f. The nonlinear least‐squares fitting was carried out with a modified Randles‐type equivalent circuit and the charge transfer resistance was calculated to be 4.05 and 8.44 Ω for Ca and Mg electrolytes, respectively.[[qv: 23a]] This charge transfer resistance is mainly associated with the dehydration of the ions since it is presumed that partial dehydration occurs during the insertion of hydrate Mg‐ion into Prussian blue analogues.^31^ Because of the low charge density of Ca‐ion, only two H_2_O molecules tightly bond to Ca‐ion, while that number goes to six for Mg‐ion.^24^ The relative ease of dehydration for Ca‐ion results in a smaller interfacial charge transfer resistance than that for Mg‐ion. The faster Ca‐ion diffusion could have also contributed to the faster kinetics, but likely to a lesser extent compared with the PNDIE anode considering the mainly diffusion‐controlled nature of the CuHCF electrode reaction.

Based on Ca‐ion coordination and insertion chemistries in PNDIE and Ca_0.3_CuHCF, respectively, we have assembled a full aqueous battery. The battery reactions are as follows

$$\begin{array}{c} \mathrm{Cathode:}\;{\mathrm{Ca}}_{0.3}\mathrm{Cu}{\left[{\mathrm{Fe(CN)}}_{6}\right]}_{0.66}.3.7H_{2}O\;\;↔\;\;\mathrm{Cu}{\left[{\mathrm{Fe(CN)}}_{6}\right]}_{0.66}.3.7H_{2}O\\ +\;0{\mathrm{.3Ca}}^{2+}+0.6e^{-}\;\;\;\;\;\;\;\;\;\;\;\;\;\;\;\;\;\;\;\;\;\;\;\;\;\;\;\;\;\;\;\;\;\;\;\;\;\; \end{array}$$

$$\mathrm{Anode}:\;1\mathrm{/n}{\left(C_{16}H_{8}N_{2}O_{4}\right)}_{n}+{\mathrm{Ca}}^{2+}+2e^{-}↔1\mathrm{/n}{\left(C_{16}H_{8}N_{2}O_{4}‐\mathrm{Ca}\right)}_{n}$$

$$\begin{array}{c} \mathrm{Full}\;\mathrm{cell}:\;1/n{\left(C_{16}H_{8}N_{2}O_{4}\right)}_{n}+3.33{\mathrm{Ca}}_{0.3}\mathrm{Cu}{\left[\mathrm{Fe}{\left(\mathrm{CN}\right)}_{6}\right]}_{0.66}.3{\mathrm{.7H}}_{2}O\;\;↔\\ 3\mathrm{.33Cu}{\left[{\mathrm{Fe(CN)}}_{6}\right]}_{0.66}.3.7H_{2}O\;+\;1\mathrm{/n}{\left(C_{16}H_{8}N_{2}O_{4}‐\mathrm{Ca}\right)}_{n}\;\;\;\;\; \end{array}$$

The typical cyclic voltammograms of the two individual electrodes (Ca_0.3_CuHCF and PNDIE) at a scan rate of 1 mV s^−1^ are displayed in **Figure** **5**a. The redox potentials of both cathode and anode are located within the electrochemical window of the electrolyte. PNDIE shows two pairs of redox peaks at −0.44/−0.19 and −0.65/−0.45 V versus Ag/AgCl, corresponding to a two‐step two‐electron transfer during calciation/decalciation of each unit formula. Ca_0.3_CuHCF also shows well‐defined redox peaks at 0.83/0.80 V versus Ag/AgCl. Considering the average potentials of the redox peaks for both anode and cathode, we could expect a 1.24 V average output voltage of the battery. The specific energy of this battery calculated from the theoretical capacity of anode (183 mAh g^−1^) and cathode (65 mAh g^−1^) and the average voltage of ≈1.24 V is 60 Wh kg^−1^. We assembled a balanced full cell with an anode/cathode mass ratio of 1:3 considering three times as large specific capacity of PNDIE as that of Ca_0.3_CuHCF (Figure 5b). The resulting battery delivers a specific capacity of ≈40 mAh g^−1^ based on the weight of active materials on both electrodes and shows a sloping voltage profile with an average discharge voltage of ≈1.2 V at 1C (40 mAh g^−1^), corresponding to an actual specific energy of 54 Wh kg^−1^, close to that of current aqueous batteries for grid‐storage applications.^32^ This battery was cycled with at a current rate of 10C for 1000 cycles with a capacity retention of 88% (Figure 4d). The coulombic efficiency after initial 50 cycles ranged between 99.3 and 99.9%. We observe the aqueous Ca‐ion battery undergoes a stabilization process during initial cycles. This may be due to the residual oxygen dissolved in the electrolyte during the fabrication process, which becomes diminished after initial cycles. In the lower C‐rate testing at C/5 where the side reaction could become more pronounced, the capacity retention is 88% after 50 cycles (Figure S11, Supporting Information). Table S4 (Supporting Information) summarizes this work with previously reported aqueous multivalent batteries. Compared to other aqueous multivalent batteries listed in Table S4, the Ca‐ion battery shows superior discharge voltage, specific energy, and cycling stability than Mg^2+^ and Al^3+^ batteries, but is inferior to Zn^2+^ batteries simply because the high‐capacity and low‐potential Zn metal anodes could be used in aqueous Zn‐ion batteries.

**Figure 5:**
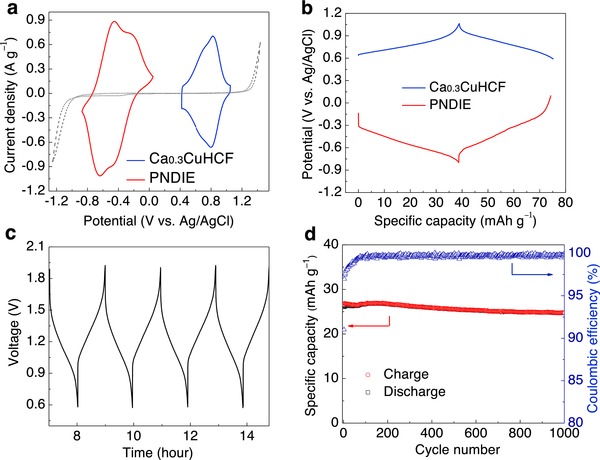
Electrochemical performance of the aqueous Ca_0.3_CuHCF//PNDIE battery. a) CV curves of PNDIE anode and Ca_0.3_CuHCF cathode in 2.5 m Ca(NO_3_)_2_ at a scan rate of 1 mV s^−1^. The dashed line represents the electrochemical stability of the electrolyte. b) The galvanostatic profiles of PNDIE and Ca_0.3_CuHCF electrodes versus Ag/AgCl reference electrode. c) Voltage profile of Ca_0.3_CuHCF//PNDIE battery at a current rate of 450 mA g^−1^ (1C, based on the total active mass). d) The cycling performance of Ca_0.3_CuHCF//PNDIE battery at 400 mA g^−1^ current density. (The capacity and the current density of the battery were calculated based on the weight of PNDIE plus Ca_0.3_CuHCF. The cutoff voltage is 0.5–1.9 V).

In summary, a new low‐cost and safe battery has been demonstrated using Ca‐ion as the charge carrier. The choice of Ca‐ion provides the advantage of any multivalent ion batteries while offers faster electrode kinetics than, for example, a Mg‐ion‐based analogue. The polyimide PNDIE anode undergoes a two‐step two‐electron enolization reaction during Ca^2+^ storage, delivering a high capacity of 160 mAh g^−1^ at −0.45 V versus Ag/AgCl with pseudocapacitor‐like high‐rate capability. The open‐framework CuHCF cathode utilizes the high‐potential Fe^2+^/Fe^3+^ couple and accommodates Ca‐ions at 0.72 V versus Ag/AgCl with a small structural strain. Both electrodes show faster electrode kinetics in a Ca‐ion electrolyte than in a Mg‐ion one as the result of the smaller size and more facile dehydration of hydrated Ca‐ions. A balanced full cell exhibits a specific energy of 54 Wh kg^−1^ at 1C rate and outstanding cycle life at both high and low current density. This novel Ca‐ion battery features nontoxic, low‐cost, and readily mass‐produced electrode materials. The use of nonflammable and low‐cost aqueous electrolytes makes manufacturing cost‐effective and effortless. Therefore, this battery will be attractive for grid‐related applications, including the smoothing of intermittent variations in power production associated with the integration of renewable energy.

## Conflict of Interest

The authors declare no conflict of interest.

## Supporting information

Supplementary
