# Supplementary material for: An Aqueous Ca‐Ion Battery
Source: Adv Sci (Weinh). 2017 Oct 26;4(12):1700465. doi: 10.1002/advs.201700465 (PMC5737234; doi:10.1002/advs.201700465)
Supplement: Supplementary file 1 — Supplementary [file ADVS-4-na-s001.pdf]

Copyright WILEY-VCH Verlag GmbH & Co. KGaA, 69469 Weinheim, Germany, 2017.

ADVANCED SCIENCE
Open Access

Supporting Information

for *Adv. Sci.,* DOI: 10.1002/advs.201700465

An Aqueous Ca-Ion Battery

Saman Gheytani, Yanliang Liang, Feilong Wu, Yan Jing, Hui Dong, Karun K. Rao, Xiaowei Chi, Fang Fang, and Yan Yao**

Copyright WILEY-VCH Verlag GmbH & Co. KGaA, 69469 Weinheim, Germany, 2013.

Supporting Information

An aqueous Ca-ion battery

Saman Gheytani, Yanliang Liang, Feilong Wu, Yan Jing, Hui Dong, Karun K. Rao, Xiaowei Chi, Fang Fang*, and Yan Yao*

Materials synthesis

PNDIE was prepared by a dehydration condensation reaction according to the previously reported method.[1] 1,4,5,8-naphthalenetetracarboxylic dianhydride (NTCDA) (2.15 g), ethylene diamine (EDA) (0.53 ml) and 150 ml Dimethylformamide (DMF) were mixed together and stirred under reflux overnight. The temperature was increased from 50 °C to 150 °C gradually. Then the intermediate was washed with toluene and ethanol for three times using centrifuge. After drying at 120 °C for four hours, the product was heated in argon for 11.5 hours at 350□. CuHCF nanoparticles were synthesized by a co-precipitation method.[2] 10 ml of 0.1 M $Cu(NO_3)_2$ and 0.05 M $K_3Fe(CN)_6$ aqueous solutions were simultaneously (~0.11 ml min^{-1}) added into 60 mL of DI-water dropwisely under vigorous stirring at room temperature. $FeCl_3$ and $Ni(NO_3)_2$ aqueous solution were used for FeHCF and NiHCF synthesis, respectively. After sonication of the brown precipitate for 20 min, the suspension was then filtered, washed with water, and dried under vacuum at room temperature for 48 hours. Ca_xCuHCF was prepared electrochemically in two steps. First, the CuHCF electrode was charged to 1.1 V vs. Ag/AgCl at the current of 60 mA g^{-1} and held at the potential for 1 hour to remove the small amount of potassium ions in pristine CuHCF ($K_{0.02}Cu[Fe(CN)_6]_{0.66}\cdot 3.7H_2O$). The electrode was washed with DI water and dried in air for 1 hour. Second, a new half-cell was re-assembled in a fresh aqueous Ca electrolyte and the

cathode was discharged to 0.3 V vs. Ag/AgCl at the current of 30 mA g^{-1} and held at the potential for 1 hour.

Materials characterization

The chemical structure of PNDIE was characterized by Fourier transform-infrared spectroscopy (FT-IR) through a Thermo Scientific Spectrometer (Nicolet iS5). Phase identification of as-prepared CuHCF and calciated Ca_xCuHCF powders were determined by high-energy X-ray diffraction (HRXRD) at Beamline 11-ID-C at APS. The morphology and microstructure of the as-synthesized powders were characterized by scanning electron microscopy (SEM, Gemini LEO 1525 microscopy). Combining thermogravimetric analysis (TA Instruments Q50) and Energy-dispersive X-ray (EDX) measurement has been used to obtain the chemical formula of CuHCF electrodes. First, we rely on EDX to determine the K:Cu:Fe ratio, which is found to be 0.02:1:0.66 and we derive the formula as $K_{0.02}Cu[Fe(CN)_6]_{0.66}$. yH_2O. Second, we used thermogravimetry analysis to extract the ratio of zeolitic water (Figure S7). From above results, we can derive the final formula as $K_{0.02}Cu[Fe(CN)_6]_{0.66}{\cdot}3.7H_2O$. EDX measurements were performed on a JEOL JSM 6400 SEM instrument operating at 15 kV equipped with an Octane Silicon Drift Detector (SDD). The X-ray absorption near edge structure (XANES) of the Fe K-edge and Cu K-edgy was measured in the transmission mode on the bending-magnet beamline of the Advanced Photon Source (APS, 20-BM-B). The incident beam was monochromatized using a Si(111) fixed-exit, double-crystal monochromator. Ex-situ samples at different states of charge were sealed in a Kapton tube. XANES data were processed following standard methods using the ATHENA software package.

Electrochemical characterization

The anode electrodes were prepared by pressing a paste made by mixing PNDIE powder, Super-p carbon black, and polytetrafluoroethylene in a weight ratio of 6:3:1 on the SS mesh current collectors followed by drying at 50°C. To prepare cathode electrodes, a mixture of CuHCF nano-powder, Super-P carbon black, graphite powder, and polyvinylidene fluoride (PVDF) (weigh ratio 75:10:5:10) was ground by hand, creating a homogeneously mixed powder. A slurry containing this powder mixture and 1-methyl-2-pyrrolidinone (NMP) was spread on Ti foil as the current collectors since it has a high overpotential for oxygen evolution reaction.[3] The electrodes were dried in air at 100 °C. The electrochemical investigation on the cathode and anode electrodes were performed in the three-electrode half-cell configuration using activated carbon as the counter electrode, Ag/AgCl as the reference electrode, and 2.5 M $Ca(NO_3)_2$ as the electrolyte (pH = 5.1). For the full-cell measurement, the as-prepared cathode electrodes have been fully calciated. EIS was collected with zero bias and a frequency sweep from 100 kHz to 100 mHz with an amplitude of 10 mV. All coin cells assembling in an open-air environment and a Biologic VMP3 potentiostat has been employed for all electrochemical measurements. For EDX SEM measurement, Ca_xHCF samples prepared at different states of charge and discharge (labeled A through F as shown in Figure 4a). Samples A–D were prepared by galvanostatic charging the Ca_xCuHCF electrodes to a specified state-of-charge (SOC) at C/2, and samples E–F, the Ca_xCuHCF electrodes were first charged to 1.05 V versus Ag/AgCl and then discharged to a specified depth-of-discharge (DOD) at C/2, which all followed by a 15 min relaxation at zero current. Then, all samples were washed with deionized water followed by drying in 70 °C prior to EDX measurement. A SEM EDX point scan was then used to quantify the Ca, K, Cu, and Fe concentrations for all samples in each of which three points were selected for quantitative elemental analysis. The detailed Ca/Cu, K/Cu, and Fe/Cu ratios for all the selected points in samples A–F are summarized in Table S2.

Electrolytic conductivity measurements

Electrolytic conductivity (κ) of the electrolytes was measured with a Thermo Scientific™ Orion Star™ A215 pH/Conductivity meter at room temperature. The conductivity cells consisted of a pair of platinum−iridium electrodes and a Pyrex cell body. The cell constants of a nominal value of 0.1 cm^{-1} were calibrated with two standard NaCl solutions of 12.9 and 1413 mS cm^{-1} nominal values.

Table S1. Properties of some elements: radius of the corresponding ion, hydrated ionic radius,[4] apparent dynamic hydration number,[5] standard reduction potential, polarization strength, electrochemical capacity, density and abundance in crustal rocks.[5]

	Li^{+}	Na^{+}	Ca^{2+}	Mg^{+}	Al^{3+}
Ionic Radius, Å	0.687	1.011	1.005	0.720	0.531
Hydrated Ionic Radius, Å	1.58	1.83	2.6	3.0	3.37
ADHN*	0.64±0.06	0.3±0.13	2.38±0.14	5.73±0.18	8.68±0.27
Crust abundance, ppm	20	23,000	50,000	29,000	82,000
$E°$ vs NHE, V	–3.04	–2.71	–2.87	–2.37	–1.66
Capacity, C cm^{-3}	7,400	4,000	7,400	13,800	28,900
Charge density, e $Å^{-3}$	0.54	0.23	0.49	1.28	4.55
Polarization Strength** (10^4 pm^{-2})	2.16	1.11	1.92	4.73	5.66

* ADHN, apparent dynamic hydration number is the number of tightly bound water molecules
**Polarization strength (P) is calculated as $P = q\, r^{-2}$, where q is the charge number of the cation and r is the ion radius.[6]

Table S2. Atomic ratios Fe/Cu, Ca/Cu, and K/Cu from the SEM EDX elemental analysis obtained from samples obtained at different states of discharge (labeled in Figure 4a)

Sample #	Point	Cu (atomic %)	Fe (atomic %)	Ca (atomic %)	K (atomic %)	Avg.(Fe/Cu) Std Deviation	Avg.(Ca/Cu) Std Deviation	Avg. (K/Cu), Std Deviation
Pristine	1	6.9	4.37	0	0.11	0.67 0.07009	0	0.019 0.0022
	2	8.54	5.3	0	0.13			
	3	7.84	5.86	0	0.14			
A	1	9.48	6.15	2.53	0.04	0.6394 0.0189	0.2903, 0.0185	0.0031 0.0009
	2	7.59	4.73	2.23	0.02			
	3	15.16	10.03	4.73	0.04			
B	1	14.78	9.91	2.41	0.04	0.6565 0.0130	0.1456, 0.0143	0.0032 0.0005
	2	9.63	6.30	1.33	0.03			
	3	8.08	5.21	1.12	0.03			
C	1	8.12	5.6	0.69	0.03	0.6634 0.0302	0.0887, 0.0043	0.0037 0.0006
	2	9.1	6.1	0.85	0.04			
	3	6.82	4.3	0.6	0.02			
D	1	6.8	4.53	0.1	0.04	0.6618 0.0081	0.0141, 0.0016	0.0006 0.0005
	2	5.44	3.65	0.08	0.03			
	3	6.61	4.33	0.1	0.04			
E	1	7.46	4.94	0.85	0.04	0.6399 0.0261	0.1353, 0.0144	0.0043 0.0009
	2	10.4	6.89	1.46	0.04			
	3	13.49	8.39	1.93	0.05			
F	1	15.86	10.77	4.95	0.02	0.6641 0.0218	0.2864, 0.0233	0.0026 0.0017
	2	7.43	5.01	1.98	0.02			
	3	7.48	4.78	2.1	0.03			

Table S3. Lattice parameter values determined from three plane groups for both charged and discharged electrode

Plane index	Lattice constant of $Ca_{0.3}CuHCF$ (Å)	Lattice constant of CuHCF (Å)
(200)	10.1634	10.2734
(400)	10.1581	10.2689
(600)	10.1612	10.2658
Average	10.1609	10.2693
Std. deviation	0.0022	0.0031

Table S4: Summary of battery performance of reported aqueous multivalent batteries

Battery structure Cathode/Anode	Charge carrier	Electrolyte	Average voltage (V)	Specific energy (Wh kg^{-1})	Cycling stability (capacity%@cycle number)	Ref.
α-MnO_2/Zn	Zn^{2+}	1 M $ZnSO_4$	1.3	175	67%@100	[7]
α-MnO_2/Zn	Zn^{2+}	2 M $ZnSO_4$ + 0.1 M $MnSO_4$	1.3	170	92%@5000	[8]
β-MnO_2/Zn	Zn^{2+}	3 M $Zn(CF_3SO_3)_2$ + 0.1 M $Mn(CF_3SO_3)_2$	1.3	159	94%@2000	[9]
$Zn_{0.25}V_2O_5$/Zn	Zn^{2+}	1 M $ZnSO_4$	0.8	150	80%@1000	[10]
ZnHCF/Zn	Zn^{2+}	1 M $ZnSO_4$	1.7	60	76%@100	[11]
NiHCF/ Polyimide	Mg^{2+}	1 M $MgSO_4$	0.6	33	60%@5000	[12]
$Al_{0.2}$CuHCF/ AC	Al^{3+}	1 M $Al(NO_3)_3$	0.4	13	90%@1000	[13]
$Ca_{0.3}$CuHCF/ PNDIE	Ca^{2+}	2.5 M $Ca(NO_3)_2$	1.2	54	90%@1000	This work

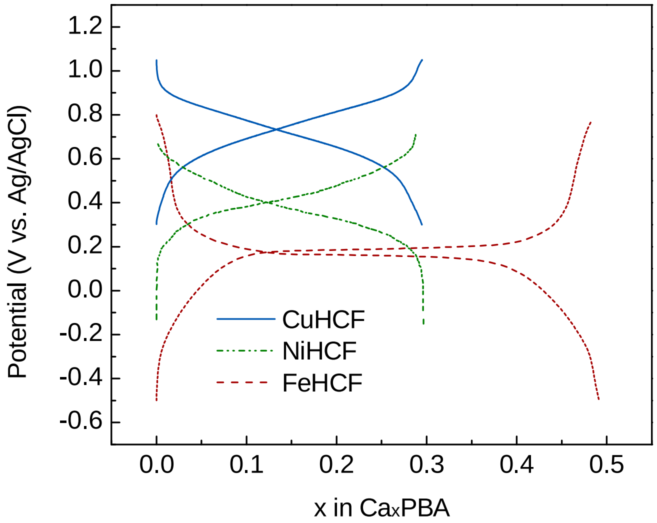

Figure S1. Charge-discharge profile of CuHCF, NiHCF, FeHCF electrodes in aqueous Ca electrolyte. We observed higher capacity for FeHCF electrode (~ 100 mAh g^{-1}) but 0.56 V lower in potential (CuHCF: 0.74 V vs. Ag/AgCl and FeHCF: 0.18 V vs. Ag/AgCl). NiHCF shows similar capacity as CuHCF (60 mAh g^{-1}) with the potential in between CuHCF and NiHCF (NiHCF: 0.35 V vs. Ag/AgCl). This data shows that CuHCF has the highest average potential while FeHCF provides higher capacity for Ca^{2+} storage. We select CuHCF as the optimum cathode materials in this study.

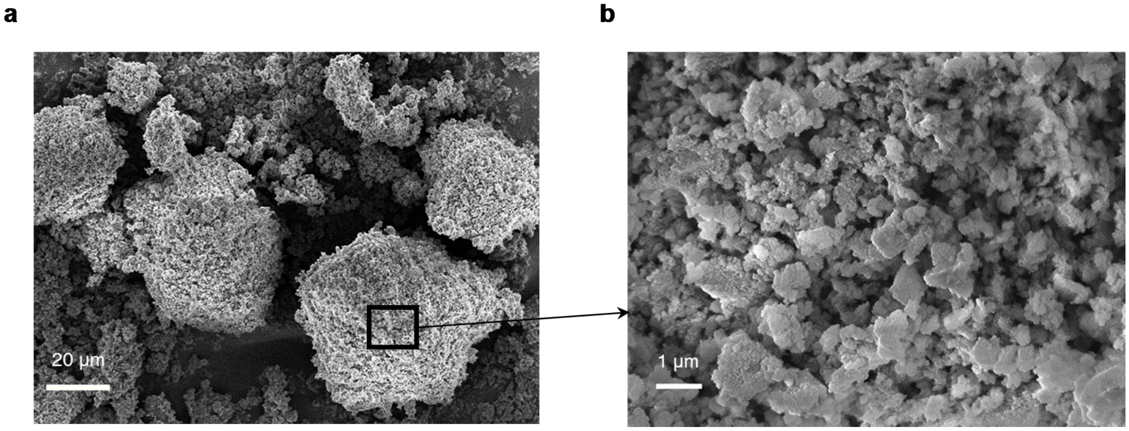

Figure S2. Morphology characterization of PNDIE powders. a) SEM images of as-synthesized PNDIE powder. b) Higher magnification of the selected area in (a) appears the PNDIE powder as the aggregated particles with an average size of less than ~1 μm.

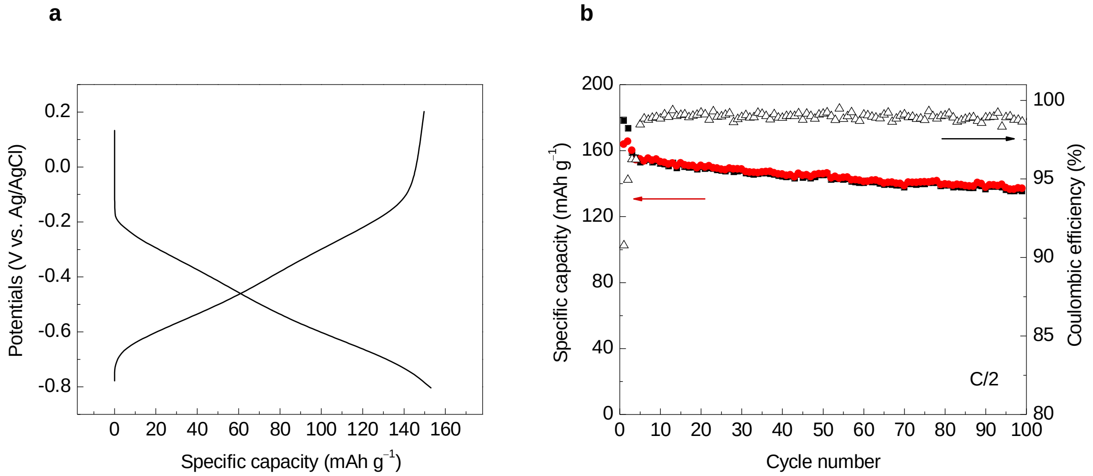

Figure S3. Low-rate performance of PNDIE electrodes in 2.5 M $Ca(NO_3)_2$ electrolyte. a) Galvanostatic Charge-discharge profile and b) cycling profiles of the PNDIE at C/2 (1C = 183 mAh g^{-1}). High coulombic efficiency of ~99% with a reversible capacity of ~153 mAh g^{-1} at a low current rate (C/2) verifies the chemical stability of PNDIE which is the result of the passivating behavior of PNDIE providing a large overpotential for hydrogen evolution reaction (HER). The high HER overpotential suppresses the locally increasing in pH on the PNDIE surface and improves its stability.[14]

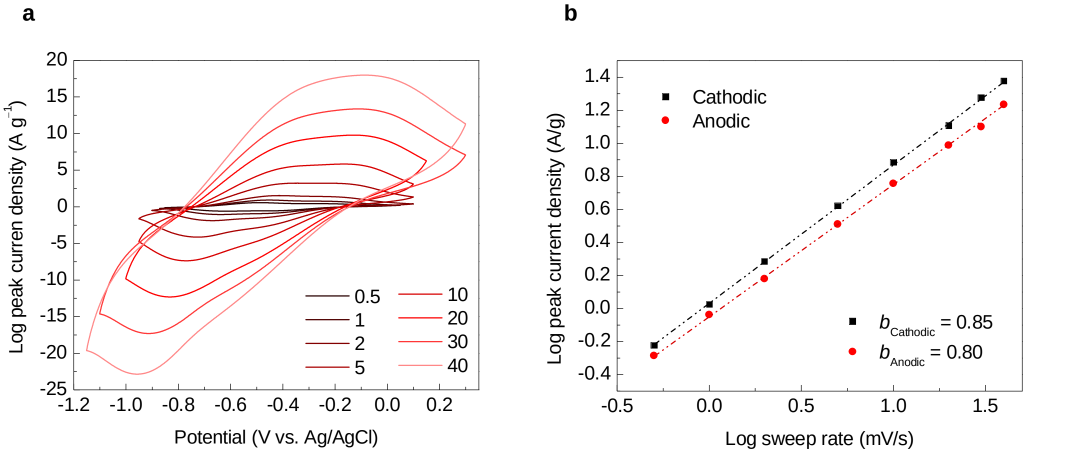

Figure S4. Kinetic study of PNDIE electrode in 2.5 M $Ca(NO_3)_2$ electrolyte. a) Cyclic-voltammetric response of PNDIE electrode to different sweep rates (v). b) corresponding logi_p versus log$v$. The peak current density and peak potential separation gradually increased as the scan rate increased but the CV curve showed an quasi-reversible behavior of PNDIE electrode at all scan rates. The relationship between the current density and scanning rate can be expressed as $i_p = a v^b$, where i_p is the peak current (A) and v is the sweep rate (mV s^{-1}), and a and b are constants. The b value of 0.5 generally indicates a diffusion controlled processes, while a value of 1.0 suggests that the reaction is a surface/adsorption charge-transfer process.[15] The b values for cathodic and anodic peaks are fitted as 0.85 and 0.80, respectively, indicating an interplay between surface- and diffusion-controlled reactions but predominantly a surface one.

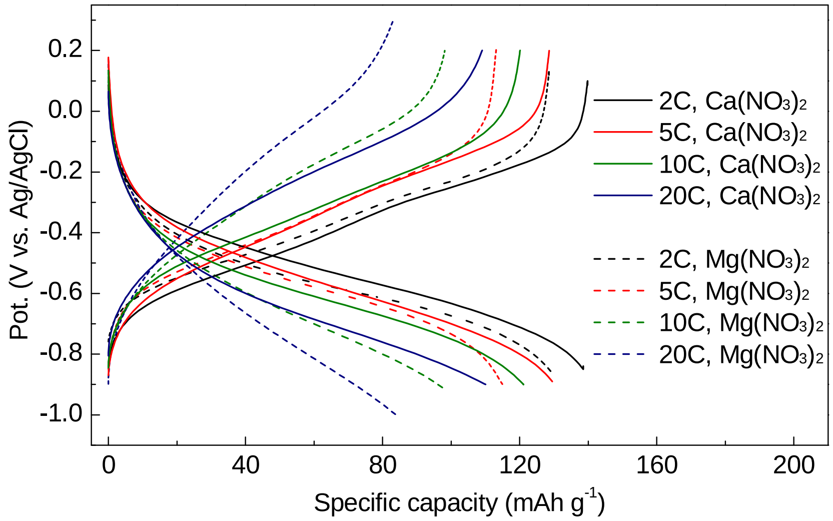

Figure S5. Galvanostatic charge-discharge profiles of PNDIE electrode in both 2.5 M $Mg(NO_3)_2$ and 2.5 M $Ca(NO_3)_2$ electrolytes at varying C-rates from 2C to 20C

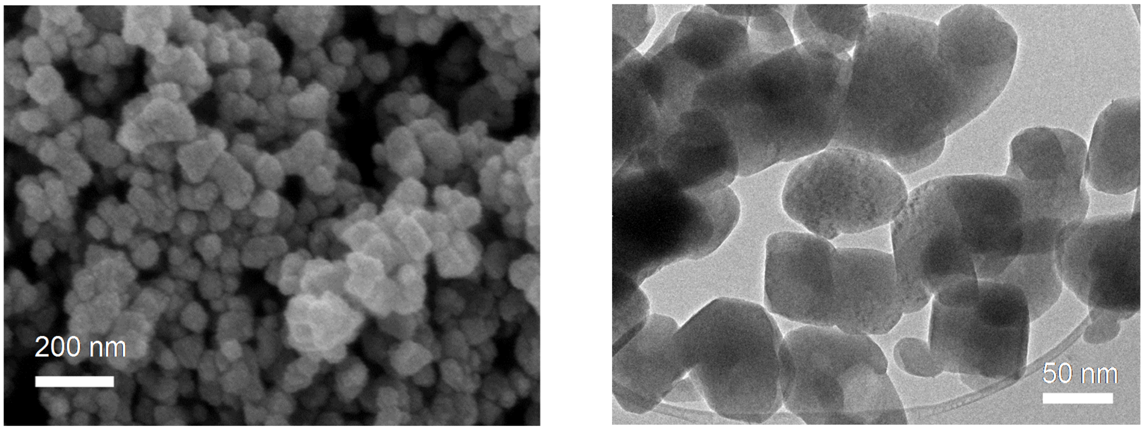

Figure S6. (a) SEM and (b) TEM images of as-synthesized CuHCF powders shows polydispersed nanoparticles of crystalline CuHCF ranging from 30 to 100 nm in diameter.

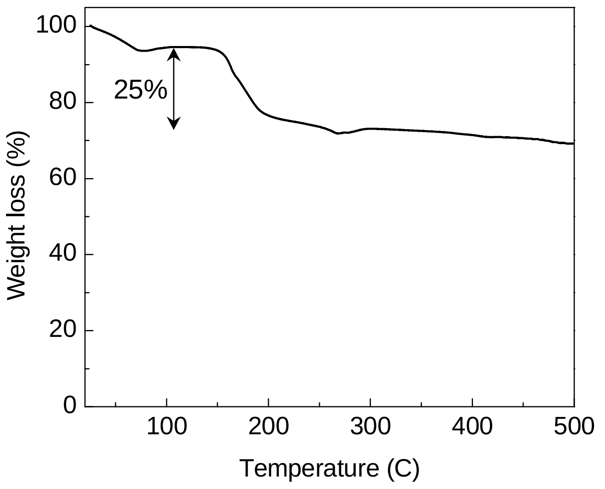

Figure S7. TGA for as-synthesized CuHCF under N_2 atmosphere at a rate of 10 °C min^{-1}.

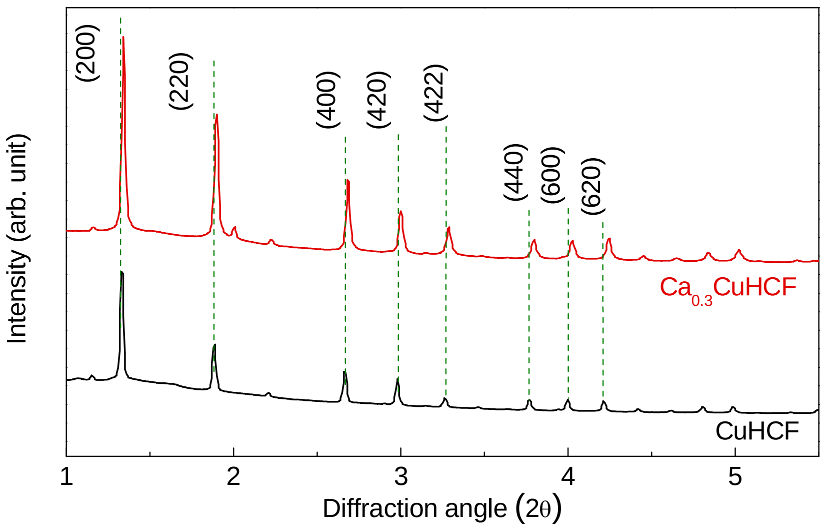

Figure S8. High-energy XRD diffraction pattern of pristine CuHCF and discharged $Ca_{0.3}$CuHCF (reduced) electrode. The X-ray wavelength was 0.1173 Å (105.1 keV)

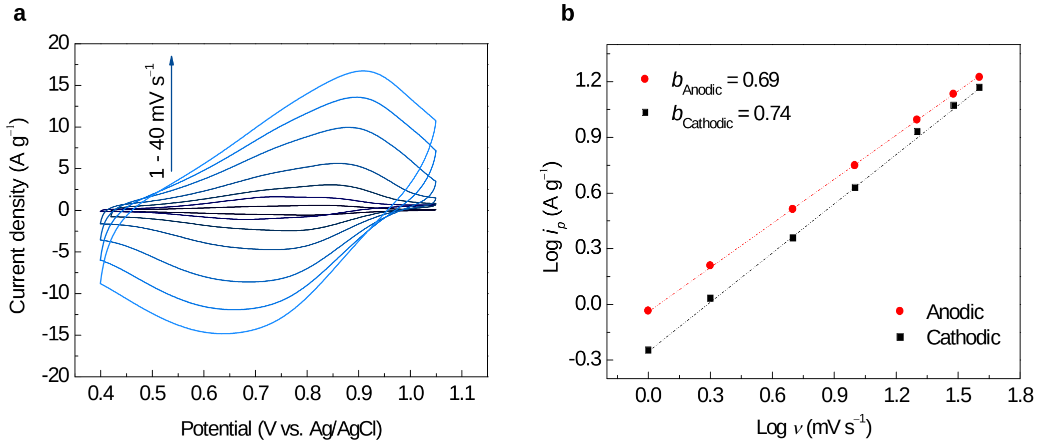

Figure S9. Kinetic study of $Ca_{0.3}CuHCF$ in 2.5 M $Ca(NO_3)_2$ electrolyte. a) CV measurement at different sweep rates. b) Corresponding logi_p versus sweep rates for cathodic and anodic peaks.

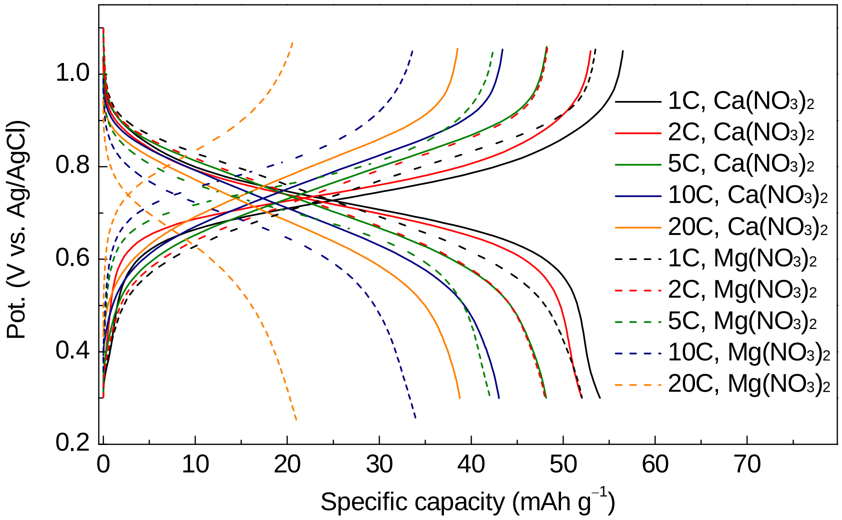

Figure S10. Charge-discharge profiles of CuHCF in 2.5 M $Ca(NO_3)_2$ and 2.5 M $Mg(NO_3)_2$ electrolytes at C-rates from 2C to 20C. The lower charge density of Ca-ion results in lower desolvation energy, therefore lower interfacial resistance.

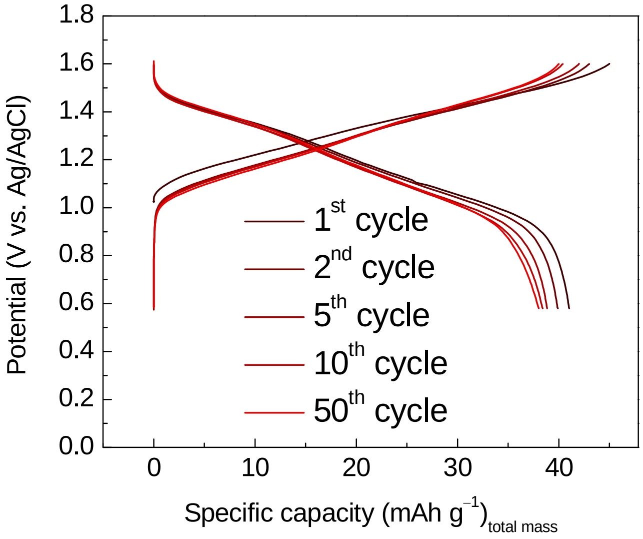

Figure S11. The galvanostatic charge-discharge of PNDIE–$Ca_{0.3}$CuHCF battery measured at a low current rate of C/5 (9 mA g^{-1}) (the capacity of the battery and current density were calculated based on the weight of PNDIE plus Ca_xCuHCF. The cutoff voltage is 0.55–1.6 V).

References

[1] R. A. Dine-Hart, *J. Polym. Sci., Part A: Polym. Chem.* **1968**, *6*, 2755.
[2] R. Y. Wang, B. Shyam, K. H. Stone, J. N. Weker, M. Pasta, H.-W. Lee, M. F. Toney, Y. Cui, *Adv. Energy Mater.* **2015**, *5*, 1401869.
[3] S. Gheytani, Y. Liang, Y. Jing, J. Q. Xu, Y. Yao, *J. Mater. Chem. A* **2016**, *4*, 395.
[4] F. David, V. Vokhmin, G. Ionova, *J. Mol. Liq.* **2001**, *90*, 45.
[5] M. Y. Kiriukhin, K. D. Collins, *Biophys. Chem.* **2002**, *99*, 155.
[6] Y. Liang, H. D. Yoo, Y. Li, J. Shuai, H. A. Calderon, F. C. Robles Hernandez, L. C. Grabow, Y. Yao, *Nano Lett.* **2015**, *15*, 2194.
[7] C. Xu, B. Li, H. Du, F. Kang, *Angew. Chem. Int. Ed.* **2012**, *51*, 933.
[8] H. Pan, Y. Shao, P. Yan, Y. Cheng, K. S. Han, Z. Nie, C. Wang, J. Yang, X. Li, P. Bhattacharya, K. T. Mueller, J. Liu, *Nat. Energy* **2016**, *1*, 16039.
[9] N. Zhang, F. Cheng, J. Liu, L. Wang, X. Long, X. Liu, F. Li, J. Chen, *Nat. Commun.* **2017**, *8*, 405.
[10] D. Kundu, B. D. Adams, V. Duffort, S. H. Vajargah, L. F. Nazar, *Nat. Energy* **2016**, *1*, 16119.
[11] L. Zhang, L. Chen, X. Zhou, Z. Liu, *Adv. Energy Mater.* **2015**, *5*, 1400930.
[12] L. Chen, J. L. Bao, X. Dong, D. G. Truhlar, Y. Wang, C. Wang, Y. Xia, *ACS Energy Lett.* **2017**, *2*, 1115.
[13] Z. Li, K. Xiang, W. Xing, W. C. Carter, Y.-M. Chiang, *Adv. Energy Mater.* **2015**, *5*, 1401410.
[14] Y. Wang, X. Cui, Y. Zhang, L. Zhang, X. Gong, G. Zheng, *Adv. Mater.* **2016**, *28*, 7626.
[15] X. Dong, L. Chen, J. Liu, S. Haller, Y. Wang, Y. Xia, *Sci. Adv.* **2016**, *2*, e1501038.
